# Cyclophilin A Associates with Enterovirus-71 Virus Capsid and Plays an Essential Role in Viral Infection as an Uncoating Regulator

**DOI:** 10.1371/journal.ppat.1004422

**Published:** 2014-10-02

**Authors:** Jie Qing, Yaxin Wang, Yuna Sun, Jiaoyan Huang, Wenzhong Yan, Jinglan Wang, Dan Su, Cheng Ni, Jian Li, Zihe Rao, Lei Liu, Zhiyong Lou

**Affiliations:** 1 Tsinghua-Peking Center for Life Sciences, Key Laboratory of Bioorganic Phosphorus Chemistry & Chemical Biology (Ministry of Education), Department of Chemistry, Tsinghua University, Beijing, China; 2 School of Medicine, Tsinghua University, Beijing, China; 3 National Laboratory of Macromolecules, Institute of Biophysics, Chinese Academy of Science, Beijing, China; 4 School of Pharmacy, East China University of Science and Technology, Shanghai, China; 5 Collaborative Innovation Center for Biotherapy, State Key Laboratory of Biotherapy and Cancer Center, West China Hospital, West China Medical School, Sichuan University, Chengdu, China; 6 Beijing No. 4 High School, Beijing, China; University of Kentucky, United States of America

## Abstract

Viruses utilize host factors for their efficient proliferation. By evaluating the inhibitory effects of compounds in our library, we identified inhibitors of cyclophilin A (CypA), a known immunosuppressor with peptidyl-prolyl *cis-trans* isomerase activity, can significantly attenuate EV71 proliferation. We demonstrated that CypA played an essential role in EV71 entry and that the RNA interference-mediated reduction of endogenous CypA expression led to decreased EV71 multiplication. We further revealed that CypA directly interacted with and modified the conformation of H-I loop of the VP1 protein in EV71 capsid, and thus regulated the uncoating process of EV71 entry step in a pH-dependent manner. Our results aid in the understanding of how host factors influence EV71 life cycle and provide new potential targets for developing antiviral agents against EV71 infection.

## Author Summary

Enterovirus 71 (EV71) is the major causative agent of hand-foot-and-mouth disease (HFMD) in Asia-Pacific region and caused over one million infection cases and nine hundred deaths in the year of 2010 in China mainland. EV71 is known to infect the young children for the sake of their undeveloped immune system. Unlike other Enterovirus (e.g. coxsackievirus), EV71 could cause severe aseptic meningitis, encephalitis, myocarditis, and acute flaccid paralysis, thus leading to high fatality rates. There is no clinically applied therapeutics. In this work, we used CypA inhibitors as bioprobes to show that CypA played an essential role in EV71 proliferation. We also elucidated the mechanism by which CypA interacted with the EV71 VP1 H-I loop and functioned as an uncoating regulator in EV71 entry step. Since there are several non-immunosuppressive CypA inhibitors, e.g. NIM-811 and Debio-025, have been reported to show antiviral potency, our results provide a potential way to discover clinical therapeutics against EV71 infection.

## Introduction

Cyclophilins (Cyps) are key cellular factors that function in numerous cellular processes, including transcriptional regulation, immune response, protein secretion, and mitochondrial function [1]. Cyps possess peptidyl-prolyl *cis*-*trans* isomerase activity and have high affinity for the immunosuppressant cyclosporine A (CsA). Cyclophilin A (CypA) is a key member of the Cyp family and was first shown to mediate the immunosuppressive function of CsA through the formation of a CsA-CypA complex. This complex binds to and inhibits the function of the phosphatase calcineurin, which normally functions to dephosphorylate NF-AT, a transcription factor important for T cell activation [1].

CypA is also known to play critical roles in the proliferation of a number of viruses, including human immunodeficiency virus type 1 (HIV-1), influenza virus, hepatitis C virus (HCV), vesicular stomatitis virus (VSV), vaccinia virus, severe acute respiratory syndrome coronavirus (SARS-CoV), rotavirus (RV) and human papillomavirus (HPV), by interacting with viral proteins or facilitating IFN-β production [2], [3]. CypA was first shown to be incorporated into HIV-1 virions through its interaction with the capsid protein (CA), and the interaction between newly synthesized HIV-1 CA and CypA is required for HIV-1 to induce dendritic cell maturation [4], [5]. CypA also interacts with other HIV-1 proteins, such as Vpr and p6, to regulate HIV infection [6], [7]. CypA was further revealed to interact with extracellular CD147, which is the main receptor for CypA on the cell membrane of human leukocytes, and this interaction can induce the phosphorylation of HIV-1 matrix protein to regulate the liberation of the reverse transcriptase complex into cytoplasm during an early stage of HIV-1 infection or can function in HIV-1 attachment to host cells [8]. But a recent research showed that CypA stabilized the HIV-1 capsid and antagonizes HIV-1 uncoating *in vitro*, indicating the versatile roles of CypA in HIV-1 infection [9]. Moreover, several lines of evidences revealed that Cyps play crucial roles in HCV life cycle. CypB was first reported to be important for HCV replication [10], but later studies showed that CypA, but not CypB, was required for HCV infection *in vitro* [11]–[14]. CypA was reported to function in the replication of HCV by increasing the affinity of the HCV polymerase NS5B for viral RNA to enhance HCV replication [13], or by binding to the HCV NS5A protein to aid in viral replication [15], [16]. Furthermore, Cyps were demonstrated to play an essential role in HPV infection by facilitating conformational changes in capsid proteins of HPV, resulting in exposure of the N-terminus of L2 protein, and the dissociation of L1 pentamers from recombinant HPV11 L1/L2 complexes in a pH-dependent manner [3], [17].

Enterovirus-71 (EV71), a member of the *Picornaviridae* family, is one of the major causative agents of hand-foot-and-mouth disease (HFMD) in pan Asia-Pacific region and results over eight millions of infections and three thousands of dead cases since 2008 [18], [19]. The genome of EV71 contains a single-stranded, positive-sense RNA (+ssRNA) and encodes a polypeptide with a molecular weight of approximate 250 kDa [20]. This polyprotein is initially processed into one structural (P1) and two non-structural (P2 and P3) regions and then undergoes proteolytic cleavage into various precursors, ultimately resulting in 11 mature proteins. Among them, P1 is further proteolyzed into VP1 to VP4 to form the viral capsid, while P2 and P3 are processed into replicase proteins. For a productive infection, virions must uncoat and release viral genome into host cytoplasm, following the successful bindings with functional receptors. Enteroviral uncoating process involves sequential capsid alterations by conformational changes [21]. During uncoating, mature particles with sediment coefficient of 160S are converted to the uncoating intermediate A particles with sediment coefficient of 135S, and subsequent empty 80S particles representing the final production of the entry process [22]. The 80S particles are empty particles that have shed genomic RNA, whereas the 135S particles retain the full complement of genomic RNA but lack some or all of their content of VP4 and have externalized most of the N-terminal extension of VP1 that is normally inside the virions [22].

The involvement of host cellular factors plays essential roles in virus proliferation. However, the knowledge of how EV71 utilizes host factors in its life cycle is limited. Only two extracellular membrane proteins, human P-selectin glycoprotein ligand-1 (PSGL-1) [23] and scavenger receptor B2 (SCARB2) [24], [25], as well as heparan sulfate (HS) [26], were recently identified as functional receptors for EV71 infection. Another result suggests that the binding of EV71 to human annexin II on the cell surface enhanced viral entry and infectivity, especially at a low infective dose [27]. Interestingly, SCARB2 was reported to be the exclusive uncoating receptor to trigger conversion of 160S particles to other forms during uncoating process at acidic condition, resulting in the releasing of viral genome [21]. Here we used CypA inhibitors as bioprobes to show that CypA played an essential role in EV71 proliferation. We also elucidated the mechanism by which CypA interacted with and modified the conformation of EV71 VP1 H-I loop, and thus regulated the uncoating process of EV71 entry. This CypA-EV71 capsid functional association not only provides information to understand the cellular factors used in EV71 infection, but also presents a new promising potential for the development of antiviral therapeutics.

## Results

### CypA inhibitors suppress EV71 multiplication

**Figure 1:**
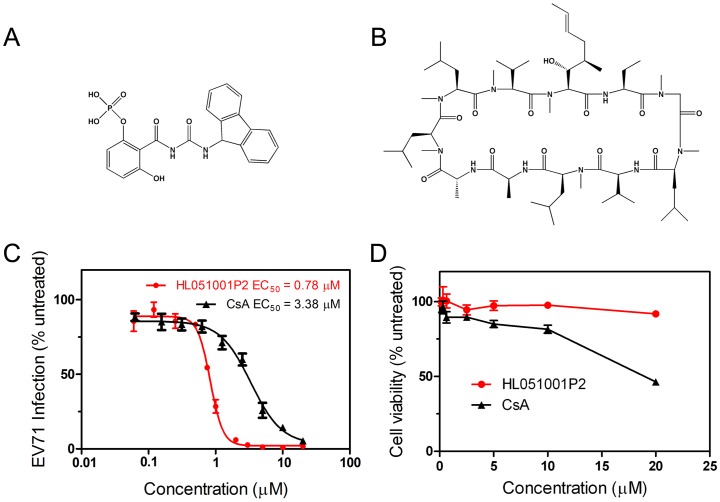
The concentration-dependent reduction of EV71 RNA following treatment with compound HL051001P2 and CsA. Chemical structures of compound HL051001P2 **(A)** and CsA **(B)**. After the RD cells were incubated for 6 h with various concentrations of compound HL051001P2 (0.06 to 20 µM for anti-EV71 activity and for cytotoxicity) and CsA (0.06 to 20 µM for anti-EV71 activity and for cytotoxicity), the cells were then infected with EV71 virus at an MOI of 1. **(C)** The infection of RD cells with EV71 virus in the presence of the compound treatments were quantified by qRT-PCR at 24 hpi, and the data were expressed as the percentage of EV71 RNA from the untreated control cells; the mRNA level of GAPDH was used as an internal control. Each data point represents the average of three replicates. Error bars represent the SEM. **(D)** To monitor the cytotoxic effects, the viability of the RD cells was determined after compound treatment by using a WST-1 based assay, which was compared with that of the untreated control cells at 24 h post compound treatment. Each data point represents the average of three replicates. The EV71 virus used in these experiments was strain AnHui1.

Our compound collection, which includes 950 chemically synthesized compounds, was screened by using rhabdomyosarcoma (RD) cells infected with the EV71 virus strain AnHui1. This screen identified compound HL051001P2 (Figs. 1A) as a potent inhibitor of viral proliferation, with an EC_50_ value of 780 nM, by measuring EV71 virus RNA through quantitative RT-PCR (qRT-PCR) (Fig. 1C). No significant cytotoxicity was observed from compound HL051001P2 at concentrations below 20 µM, as demonstrated by the WST-1-based assay (Fig. 1D), indicating that the inhibition of EV71 proliferation was specific.

Because compound HL051001P2 was previously reported to function as a CypA inhibitor [28], we next selected CsA (Fig. 1B), a well-known Cyp inhibitor and clinical immunosuppressant drug with antiviral effects, to suppress HIV-1 and HCV replication and to check whether other CypA inhibitor can also inhibit EV71 replication. The results revealed that CsA clearly impaired EV71 proliferation with an EC_50_ value of 3.5 µM (Fig. 1C); however, CsA had a slightly higher cytotoxicity than HL051001P2 (Fig. 1D). Because Cyps are known to be involved in the viral life cycle, our interest in identifying host factors in the EV71 life cycle and antiviral agents prompted us to initiate further investigations to study the working mechanism of CypA in the EV71 life cycle and the inhibitory mechanism by which Cyp inhibitors block EV71 replication.

### CypA plays a critical role in EV71 virus proliferation

Over ten subfamilies have been identified in the Cyp family to date, among which CypA and CypB are the most abundant subtypes [10]. To clarify which type of Cyp is most essential for EV71 proliferation, we next used the RNA interference (RNAi) method to investigate the impact of CypA or CypB on EV71 virus proliferation.

**Figure 2:**
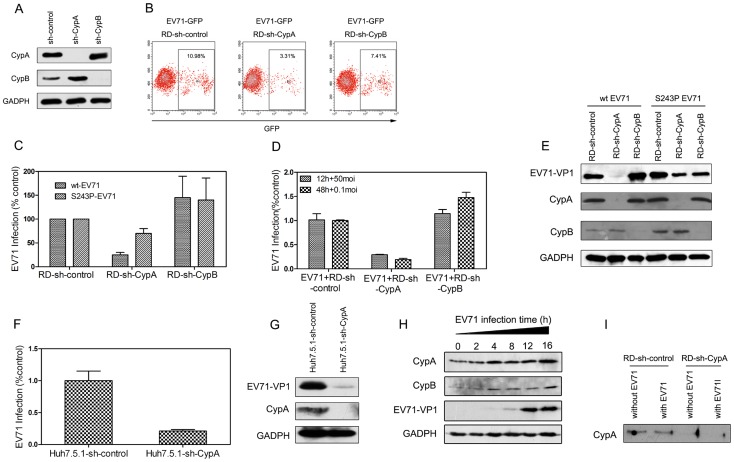
CypA regulated EV71 proliferation. **(A)** The knockdown of endogenous CypA and CypB proteins. RD cells were infected with shRNA recombinant lentiviruses that were specific for CypA (sh-CypA), CypB (sh-CypB), or with a randomized shRNA (sh-control). At 72 hpi, CypA (top), CypB (middle), and GAPDH, (an internal control) (bottom), were detected in the total cell lysates by immunoblotting analysis. **(B)** Flow cytometric studies of the RD-sh-control, RD-sh-CypA, and RD-sh-CypB cells that were infected with the EV71-GFP virus at an MOI of 0.5 after 24 h. The frames indicate the virus-infected cells, and the ratio of the infected cells is indicated in the inset. **(C)** Quantifying the infection of the RD-sh-control, RD-sh-CypA, and RD-sh-CypB cells infected with wt- or S243P-EV71 at an MOI of 1 by qRT-PCR. **(D)** Quantifying the infection of the RD-sh-control, RD-sh-CypA, and RD-sh-CypB cells infected with wt-EV71 at MOIs of 0.1 and 50 after 12 h and 48 h of infection, respectively. The data were expressed as the percentage of EV71 RNA from the untreated control cells; the mRNA level of GAPDH was used as an internal control. Each data point represents the average of three replicates. The error bars represent the SEM. **(E)** Detecting the EV71-VP1 protein expression when RD-sh-control, RD-sh-CypA, and RD-sh-CypB cells were infected with wt-EV71 or S243P-EV71 virus at an MOI of 1 after 24 h by immunoblotting analysis. **(F)** Quantifying the infection of the Huh7.5.1-sh-control and Huh7.5.1-sh-CypA cells that were infected with wt-EV71 at an MOI of 10 by qRT-PCR. **(G)** Detecting the EV71-VP1 protein expression when Huh7.5.1-sh-control and Huh7.5.1-sh-CypA cells were infected with wt-EV71 at an MOI of 10 by using immunoblotting analysis. **(H)** Detecting the expression of CypA and CypB in RD cells at different time points following infection with wt-EV71. **(I)** Detecting the existence of secreted CypA in supernatants from the RD-sh-control and RD-sh-CypA cells infected with wt-EV71. The expression levels of CypA, CypB, EV71-VP1 and GADPH were examined by immunoblotting analysis.

We first introduced short hairpin RNAs (shRNAs) that were designed to recognize the 3′ non-coding region of CypA (sh-CypA) or CypB (sh-CypB) by lentiviral vectors into RD cells to downregulate endogenous CypA and CypB expression [12]. The expression of glyceraldehyde-3-phosphate dehydrogenase (GAPDH), a housekeeping gene used as an internal control, was not downregulated (Fig. 2A). We obtained stable knockdown cell lines through resistance gene screening and then infected these shRNA-RD cells by applying an EV71-GFP virus with a multiplicity of infection (MOI) of 0.5. The results of the flow cytometric studies revealed that 10.98% of the RD cells with negative control shRNA (RD-sh-control) were infected with the EV71-GFP virus, and the infection ratio was decreased to 3.31% in the RD-sh-CypA cells. However, the infection rate remained at 7.41% in the RD-sh-CypB cells, suggesting that CypA dominantly impacted EV71 infection (Fig. 2B).

Moreover, when infected with EV71 at a multiplicity of infection (MOI) of 1, the EV71 RNA level in the CypA knockdown cells was diminished to approximately 20% of the levels observed in the RD-sh-control cells, whereas the CypB knockdown did not result in this reduction (Fig. 2C). We also infected the RD-sh-CypA and RD-sh-CypB cells with EV71 at MOIs of 0.1 and 50, respectively, and the results revealed a similar finding as the one caused by EV71 infection at an MOI of 1 (Fig. 2D). Furthermore, the expression level of EV71 VP1 protein, which is the major component of the EV71 capsid [18], [29], was significantly reduced by the knockdown of CypA, but not CypB, which is consistent with the impact of CypA reduction on the EV71 RNA level (Fig. 2E, left half). An interesting observation is that the CypB knockdown resulted in a small increase in the EV71 RNA replication and VP1 expression (Figs. 2C and 2E).

To verify the impact of CypA in the different cell lines used for EV71 infection, we generated an additional stable CypA knockdown Huh7.5.1 cell line (Huh7.5.1-sh-CypA) and observed the EV71 infections (at MOI of 10) in Huh7.5.1-sh-CypA and Huh7.5.1 cells with the negative control shRNA (Huh7.5.1-sh-control). The results demonstrated that the decreased EV71 RNA replication (Fig. 2F) and VP1 expression (Fig. 2G) were similar to those in the RD cell lines. Taken together, all these data suggested that the loss of CypA function is specifically associated with the inhibition of EV71 proliferation.

Previous studies suggested that CypA was upregulated during virus infection and is correlated with the final results of the infection [2], [30], [31]. Similarly, the expression of host cell CypA was upregulated in the RD cells following EV71 infection (Fig. 2H). We also found that EV71 infection led a very minor alteration of secreted CypA in the supernatant of RD-sh-control cells, and very little CypA could be detected in the RD-sh-CypA culture with or without EV71 infection (Fig. 2I). This finding indicated that CypA was present in host cells, but not those that were secreted in the supernatant, and CypA is upregulated following EV71 infection.

### Resistance to compound HL051001P2 maps to the VP1 protein

CypA was found to interact with different viral proteins and affect different stages of the viral life cycle through distinct mechanisms [2]. To define the working target of CypA in the EV71 virus, we generated an EV71 virus that was resistant to a CypA inhibitor through multiple cell culture passages in the presence of compound HL051001P2.

Sequence analyses of the entire genome of multiple resistant viruses identified only a single T-to-C mutation at nucleotide position 3,164 of the EV71 genome (Table S1). This mutation translated into a single amino acid substitution of a serine to a proline at residue VP1-243 (all residue numbers correspond to the residue in the sequence of the VP1 protein, not in the polypeptide), which is located in the H-I loop of VP1 [32].

To confirm that the VP1-S243P mutant was the mutation that contributed to the resistant phenotype of the selected mutant virus, we engineered a recombinant mutant EV71 virus (S243P-EV71) from the wild type EV71 (wt-EV71) strain AnHui1 through the introduction of a single serine-to-proline substitution at the VP1-243 position and investigated the sensitivity of the S243P-EV71 virus to compound HL051001P2 or CsA in comparison with wt-EV71. The EC_50_ value of compound HL051001P2 on S243P-EV71 proliferation (3.56 µM) was approximately 5-fold higher than the EC_50_ value for wt-EV71, and the EC_50_ value of CsA was 2-fold higher for the S243P mutated virus (Table S2).

Moreover, following an infection with the drug-resistant S243P-EV71 recombinant virus, the EV71 RNA level in the RD-sh-CypA cells was approximately 70% of the levels in the RD-sh-control cells. This value was much higher than the values observed in RD-sh-CypA cells that were infected with wt-EV71 virus (approximately 25%), suggesting that the VP1-S243P mutant rescued EV71 replication. The expression level of EV71 VP1 protein also consistently recovered to normal levels following infection with the S243P-EV71 virus (Fig. 2E, right half). These data again revealed that the VP1-S243P mutant is specifically associated with resistance to the CypA inhibitor.

### CypA interacts with the H-I loop of VP1 in the EV71 capsid

CypA has peptidyl-prolyl *cis*-*trans* isomerase activity to facilitate the conformational modification of proline residue. By examining the protein sequence of EV71 VP1, we found that there is only one proline residue located close to the VP1-S243 position, i.e., VP1-P246. We hypothesized that the resistant mutation from a serine residue to a proline residue at the VP1-243 position may increase the binding affinity of VP1 to CypA and help the virus escape from drug treatment and the depletion of the endogenous CypA. To clarify the mechanisms underlying the CypA regulation of the EV71 life cycle, we analyzed the molecular interaction of CypA with EV71 virions or the VP1 H-I loop by using a GST pull-down assay.

**Figure 3:**
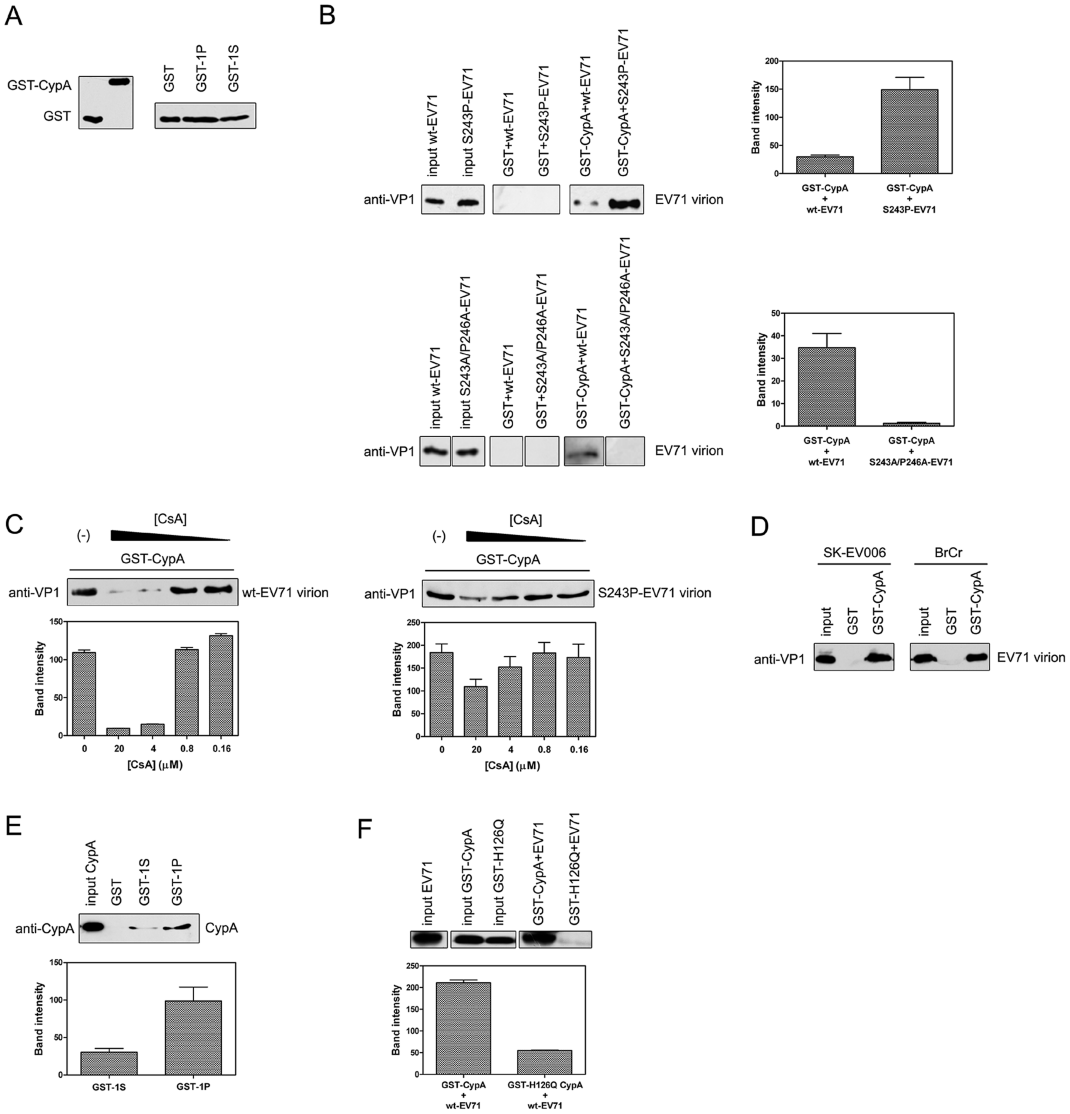
CypA interacted with the EV71 virion and the VP1 H-I loop. **(A)** Inputs that were used in the GST-pulldown assays. **(B)** The S243P-EV71 virion (upper panel) exhibited a higher, but S243A/P246A-EV71 (bottom panel) showed weakened binding affinity to CypA than the wt-EV71 virion (strain AnHui1). The bands indicating the interactions between GST-CypA with wt-, S243P-, or S243A/P246A-EV71 virions were quantified. Each column represents the average of three replicates. Error bars represent the SEM. **(C)** The CypA interaction with the EV71 virion (strain AnHui1) was disrupted by CsA in a dose-dependent manner, and the S-to-P substitution rescued this interaction. A GST pulldown assay between the GST-CypA and wt-EV71 or S243P-EV71 virions was performed in the absence (lane 1) or presence (lanes 2–5) of CsA. The CsA concentrations in lanes 2–5 are 20, 4, 0.8, and 0.16 µM, respectively. Similar amounts of wt- and S243P-EV71 viruses and GST-tagged CypA were loaded into the GST-pulldown assay. The bands were quantified and each column represents the average of three replicates. Error bars represent the SEM. **(D)** CypA was bound to different EV71 virions. SK-EV006 refers to the SK-EV006-LPS1 strain, and BrCr refers to the BrCr strain. **(E)** The mutation at position 243 increases the interaction of CypA with the H-I loop of EV71 VP1. All experiments were performed at least three times. The sequence of the 1S peptide is GSSKSKYPL, and the sequence of the 1P peptide is GSSKPKYPL. The bands for the wt-EV71 interactions with GST-1S or GST-1P were quantified. Each column represents the average of three replicates. Error bars represent the SEM. **(F)** A catalytic-defective mutant of CypA called H126Q significantly attenuated the binding of wt-EV71 virions to CypA. The bands for the interactions between wt-EV71 and CypA or the CypA mutant were quantified. Each column represents the average of three replicates. Error bars represent the SEM. The bands were quantified with ImageJ software.

We first checked whether recombinant CypA protein could associate with the EV71 virions (Fig. 3). By using a GST-tagged CypA as a probe (Fig. 3A), we demonstrated that recombinant CypA protein was clearly bound to wt-EV71, and the interaction of CypA with the EV71 virion was increased by substituting with a proline residue at VP1-S243 (Fig. 3B, upper panel). However, when we replaced both S243 and P246 with alanine residues, the mutated virus, i.e., S243A/P246A-EV71, cannot bind with CypA (Fig. 3B, bottom panel). This interaction between CypA and the EV71 virion was also reduced in a dose-dependent manner after treating with CsA and can almost be abolished at a concentration of 4 µM CsA (Fig. 3C, upper panel). Additionally, the S243P mutant rescued the interaction between CypA and EV71 under CsA treatment (Fig. 3C, bottom panel). We further demonstrated that recombinant CypA protein not only bound to wt-EV71 strain AnHui1 but also to other strains of the wt-EV71 virus (Fig. 3D).

We next fused the peptide of the H-I loop (239-GSSKSKYPL-247) (GST-1S) and the H-I loop with the S243P substitution (239-GSSKPKYPL-247) (GST-1P) to the GST tag as probes (Fig. 3A) to test whether CypA can directly bind to the VP1 H-I loop *in vitro*. The result demonstrated that CypA directly interacted with the H-I loop of EV71 VP1, and the mutation of the serine at the VP1-243 position to proline can clearly increase the binding affinity of the H-I loop for CypA (Fig. 3E). We also used NMR spectra to demonstrate that recombinant CypA binding to chemically synthesized EV71 VP1 H-I loop peptides caused chemical shift changes, suggesting that CypA catalyzed the correct *cis*-*trans* reaction of the VP1 H-I loop (Fig. S1). Moreover, a reported catalytic-defective mutant of CypA called H126Q [33] eliminated the interaction between CypA and EV71 virions (Fig. 3F). A similar attenuation of the interaction between the CypA H126Q mutant and virions was also reported in HIV-1 and HCV [12], [34]. Taken together, all these results revealed that CypA functioned directly at the H-I loop of EV71 VP1, and the replacement of serine with proline at the VP1-S243 position could increase the binding affinity of CypA with EV71 virions.

### CypA functions in the entry step of EV71 infection

S243 is located in the H-I loop of the VP1 protein of the EV71 virus, and several loop regions of the VP1 protein are known to play critical roles in the entry step during picornavirus infection [35]. Therefore, we hypothesized that CypA may also act in the entry step of the EV71 life cycle.

**Figure 4:**
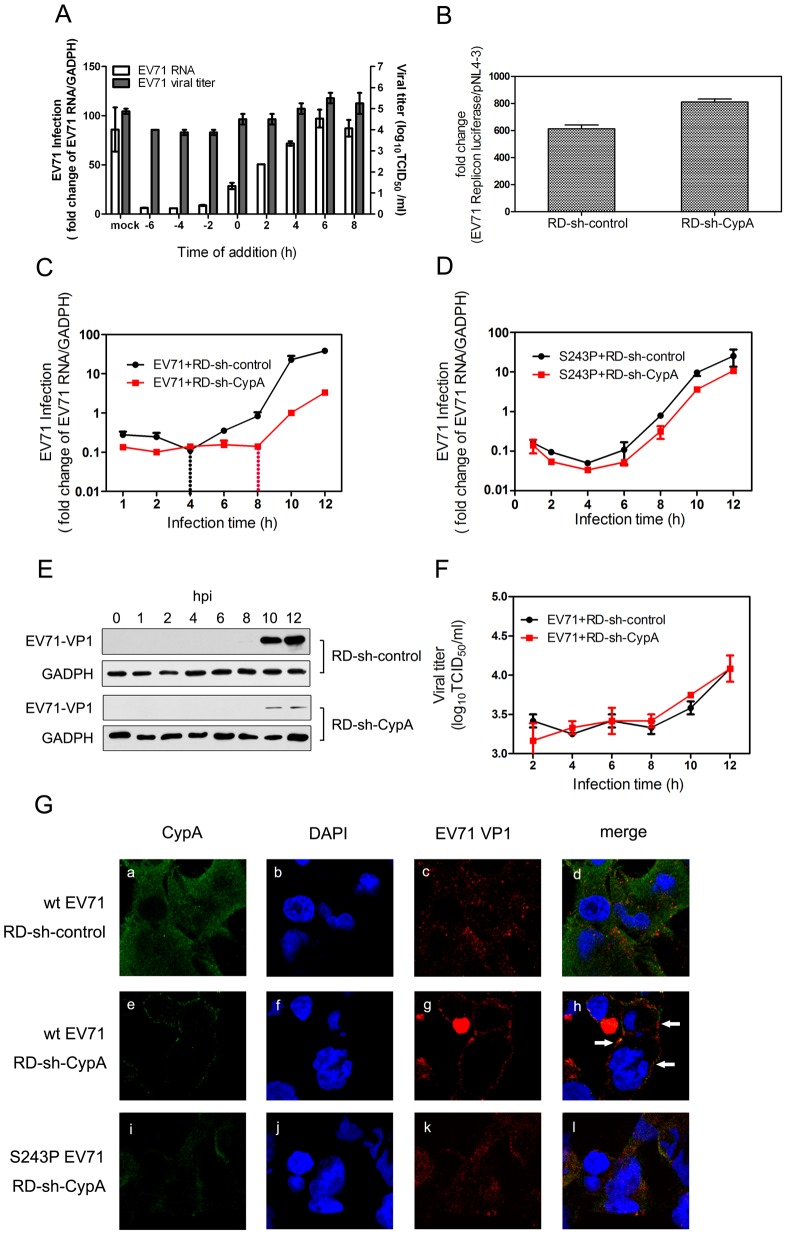
CypA functions in the entry step of EV71 infection. **(A)** The effect of adding compound HL051001P2 (5 µM) at various times during the replicative cycle of EV71. RD cells were infected by wt-EV71 virus at an MOI of 50 and the inhibitor was added at −6, −4, −2, 0, 2, 4, 6 and 8 hpi. The data for the EV71 genome were expressed as the percentage of EV71 RNA from the untreated control cells; the mRNA level of GAPDH was used as an internal control. The data for EV71 viral titers in the supernatant were measured as previously described. **(B)** The EV71 subgenomic replicon RNA was transfected into RD-sh-control and RD-sh-CypA cells, and the luciferase levels were quantified by measuring the firefly luciferase activity in relative luminescence units at 24 hpi; the firefly luciferase activity of pNL4-3 was used as an internal control. The growth curves of wt-EV71 **(C)** and S243P-EV71 **(D)** in RD-sh-control or RD-sh-CypA cells at an MOI of 50. The EV71 infection was quantified by using qRT-PCR to detect the EV71 RNA in the RD-sh-control or RD-sh-CypA cells at different times post infection, and the mRNA level of GAPDH was used as an internal control. The data represent the means of three independent experiments. Error bars represent the SEM. **(E)** The EV71 VP1 expression was also checked in the EV71-infected RD-sh-control and RD-sh-CypA cells by immunoblotting analysis. **(F)** Viral titers in the supernatant were measured at different times post infection. **(G)** The subcellular localization of CypA and EV71. Immunofluorescence analysis was performed on RD-sh-control and RD-sh-CypA cells infected with wt- or S243P-EV71 at an MOI of 100. At 2 hpi, the cells were fixed and stained with anti-CypA (panels a, e and I, green) or anti-EV71 VP1 (panels c, g, and k, red) antibodies, and DAPI was used to visualize the nuclei (panels b, f, and j, blue). Panels a-d, e-h, and i-l show the same cells. The merged images are shown in panels d, h, and l, respectively.

To verify this hypothesis, we first infected RD cells with EV71 and treated them with 5 µM compound HL051001P2 at −6, −4, −2, 0, 2, 4, 6 and 8 h post-infection (hpi), in which 0 hpi indicates the supply of a virus infection inhibitor. The results showed that the inhibition of EV71 by HL051001P2 represented a clear dependence on the treatment time. The HL051001P2 treatments at −6 to 0 hpi showed the inhibition of EV71 replication, whereas the anti-EV71 effect of the treatments after 2 hpi was significantly attenuated (Fig. 4A). We further transfected an EV71 subgenomic replicon RNA lacking the P1 region in the RD-sh-control and RD-sh-CypA cells and found that the EV71 RNA replication inside the host cells was not affected by the downregulation of CypA (Fig. 4B). These results indicated that CypA affected the early step, but not genome replication, during EV71 infection.

By detecting viral RNA at different time points in RD cells that had been infected with the EV71 virus, we found that the amount of EV71 RNA in the RD-sh-CypA cells decreased to less than 50% of that in the RD-sh-control cells at 1 hpi (Fig. 4C, start point), and this reduction was reversed when the RD-sh-CypA cells were infected with S243P-EV71 virus (Fig. 4D). When we detected EV71 VP1 expression, we found that VP1 expression can be clearly and consistently attenuated in EV71-infected RD-sh-CypA cells in comparison with EV71-infected RD-sh-control cells from 10 hpi (Fig. 4E). Moreover, the augmentation of EV71 virus RNA in RD-sh-control cells indicated that the replication of EV71 RNA began at 4 hpi (Fig. 4C, black line); by contrast, this stage was obviously delayed to 8 hpi in the RD-sh-CypA cells (Fig. 4C, red line). However, when we infected RD or RD-sh-CypA cells with S243P-EV71, the growth curves revealed a similar curve, indicating that the VP1-S243P mutant confers resistance to CypA depletion (Fig. 4D). When we checked the viral titers in the culture, we found that the viral titers in the supernatant were not clearly altered in EV71-infected RD-sh-CypA and RD-sh-control cells (Fig. 4F). We also infected RD cells by using wt-EV71 with a 5 µM HL051001P2 treatment and measured the viral titers in the supernatant at different hpis (Fig. 4A). The results showed that infectious viral production was affected by the inhibitor at -6, -4 and -2 hpi, but not as significantly as the impact on the intracellular viral genome at the same hpi. We thus speculated that CypA depletion blocked viral entry during re-infection and left more viruses in the culture.

We further examined the internalization of EV71 by using immunofluorescence (Fig. 4G). The RD-sh-control and RD-sh-CypA cells were infected with wt- and S243P-EV71, and endogenous CypA and EV71 VP1 proteins were analyzed by immunofluorescence. In the RD-sh-control cells infected with wt-EV71, EV71 VP1 was distributed throughout the cytoplasm at 2 hpi, which was indicative that the virus particle internalization and localization with CypA was random (Fig. 4G, panel a-c). The downregulation of CypA in the RD-sh-CypA cells was first confirmed (Fig. 2A). In RD-sh-CypA cells infected with wt-EV71, the localization of wt-EV71 was restricted to the cytoplasm of the perimembrane region at 2 hpi (Fig. 4G, panel d-f). We can also observe the colocalization of EV71 VP1 with CypA, suggesting that the knockdown of CypA inhibited the internalization of EV71 and CypA was accumulated around EV71 virions. By contrast, the internalization of EV71 was rescued by the S243P-EV71 mutant (Fig. 4G, panel g-i). A similar observation was also reported in the HPV16 pseudovirus in the presence of the CypA inhibitor [12]. These data indicated that CypA depletion inhibited the internalization of EV71 into the host cells.

### Treating with CypA did not enhance the binding of EV71 virions to functional receptors

**Figure 5:**
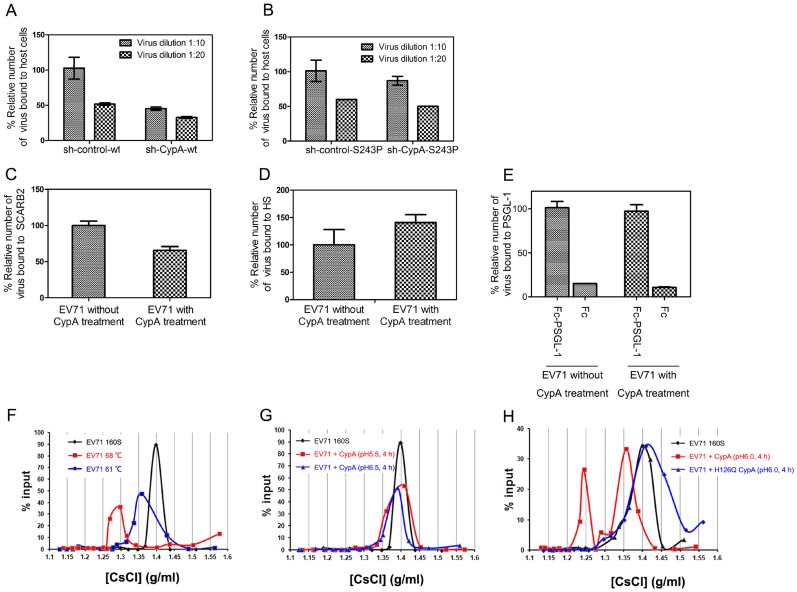
CypA is an uncoating regulator of EV71 entry. **(A)** and **(B)** The binding assay of the EV71 virions with the host cells. The binding capacity of the wt- **(A)** or S243P-EV71 viruses **(B)**. Two conditions were assessed as follows: 10-fold diluted (1∶10) and 20-fold diluted (1∶20) standardized viral stocks (10^8^ TCID_50_/ml). The amount of bound virus was measured by using a qRT-PCR assay. Error bars represent the SEM. **(C-E)** The binding of EV71 virion to reported receptors, without or with CypA treatment, including the immobilized His-tagged SCARB2 **(C)**, heparin-sepharose column **(D)**, and Fc-PSGL-1 or control Fc **(E)**. The amount of bound virus was measured by using a quantitative real-time qRT-PCR assay. Each data point for (C) and (E) represents the average of three replicates and each data point for (D) represents the average of ten replicates. Error bars represent the SEM. **(F-I)** The virion flotation assay with CsCl density gradient ultracentrifugation. Purified viruses (1×10^10^ genome copies) were used in each experiment. The samples were analyzed in a 1.1–1.5 g/ml discontinuous CsCl gradient by ultracentrifugation at 41,000 rpm for 10 h at 4°C in a SIW41Ti rotor. **(F)** Native 160S virions were treated at 61°C and 68°C under neutral conditions and analyzed by ultracentrifugation. **(G)** Native 160S virions were incubated with 20 µg of CypA followed by incubation at 37°C for 4 h at pH 5.5 and pH 6.5. **(H)** Native 160S virions were incubated with 20 µg of CypA or catalytic-defective mutant H126Q CypA followed by incubation at 37°C for 4 h at pH 6.0.

The entry of EV71 can be further divided into the following two processes: 1) receptor binding and 2) uncoating to release the viral genome [35]. To demonstrate the exact function of CypA in EV71 entry, we first checked the effect of CypA downregulation in the binding of EV71 virions to host cells. The results showed that EV71 binding to host cells was attenuated by CypA knockdown (Fig. 5A) and could be rescued by the substitution of S243 with a proline residue (from 50% attenuation to 90% attenuation) (Fig. 5B). We then checked the binding affinity of three reported EV71 functional receptors, *i.e.*, SCARB2, PSGL-1 and HS, for the wt-EV71 virions without or with CypA treatment. The results revealed that CypA treatment did not lead to obvious upregulation in the binding with EV71 functional receptors (Figs. 5C-5E). Together with the result showing that CypA directly interacts with the EV71 virion, the CypA treatment is not likely to enhance the binding to all reported receptors, and the attenuation of EV71 virions that are binding to RD-sh-CypA and compensation by S243P mutation are likely to resulted in an interaction change between the virions and CypA located at the cell membrane.

### CypA is an uncoating regulator of EV71 entry

The conversion from EV71 160S particles to 135S particles can be induced by uncoating SCARB2 under an acidic condition [21]. However, recent biochemical and structural studies have suggested that simply heating the 160S particles near 60°C induces virus expansion and RNA genome release, and heating over 65°C leads to the subsequent protein melting of virions during uncoating [36]. We next used a previously reported virion flotation assay [21], which is used to detect the conversion of 160S particles into other forms during viral uncoating, to check whether the uncoating process of EV71 entry could be affected by CypA. To be consistent, EV71 virions that were treated at 61°C exhibited a smaller shift (Fig. 5F, blue line) from the native peak (160S) (Fig. 5F, black line) after ultracentrifugation in a 1.1–1.5 g/ml discontinuous CsCl gradient, whereas the virions that were treated at 68°C exhibited a much larger shift (Fig. 5F, red line).

We next incubated 160S virions with 20 µg of recombinant CypA followed by incubation at 37°C for 4 h at pH 5.5 and pH 6.5, respectively, before subjecting them to ultracentrifugation at 41,000 rpm for 10 h at 4°C. The results revealed that CypA cannot trigger the conversion of 160S particles at pH 5.5 and pH 6.5 (Fig. 5G). On the contrary, when a mixture of 160S particles and CypA was incubated at 37°C at pH 6.0 for 4 h, the shift from 160S particles was distinct (Fig. 5H, black and red lines). Moreover, the catalytic-defective mutant of CypA, namely H126Q, was incubated with 160S particles at 37°C under pH 6.0 for 4 h, the shift in virions was completely eliminated (Fig. 5H, blue line). All these results support the idea that CypA can regulate the uncoating process of EV71 entry in a pH-dependent manner, which plays a similar role as the only EV71 uncoating receptor, or SCARB2 [21].

### The S243P mutation in EV71 VP1 decreased viral fitness but conferred CypA inhibitor resistance

**Figure 6:**
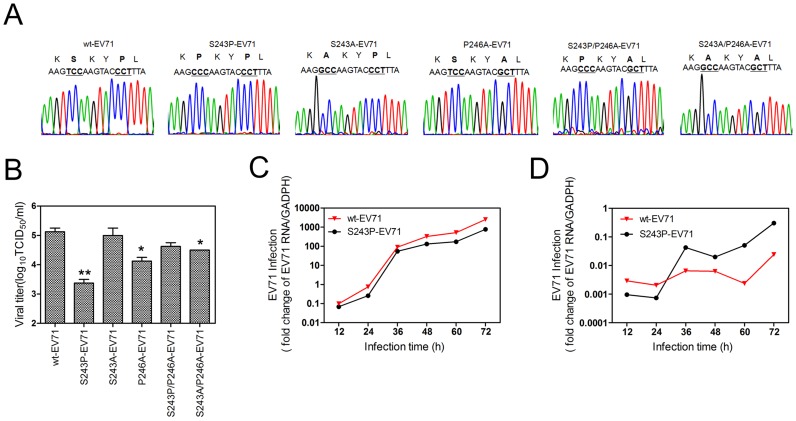
An analysis of various H-I loop mutant viruses. **(A)** A sequencing chromatogram of the mutated H-I loop region. **(B)** Viral titers in the supernatant were measured at 48 hpi after the transfection of EV71 mutant viral RNA. Each column represents the average of two replicates. Error bars represent the SEM * 0.01<P<0.05 and ** P≤0.01, by an independent sample t-test (2-tailed) when compared with wt-EV71. The growth curves of wt-EV71 and S243P-EV71 in RD cells at an MOI of 0.1 without **(C)** or with **(D)** inhibitor HL051001P2 treatment. The EV71 infection was quantified by using qRT-PCR to detect the EV71 RNA in cells at different times post infection, and the mRNA level of GAPDH was used as an internal control.

To study the fitness of the mutation in the VP1 H-I loop, we transfected RD cells with RNA transcripts of EV71 recombinants containing the 5 coding mutations and generated recombinant viruses, which were designated as S243P-EV71, S243A-EV71, P246A-EV71, S243P/P246A-EV71, and S243A/P246A-EV71 (Fig. 6). In comparison with the wt-EV71, S240A-EV71 and S240P/P246A-EV71 had almost equal supernatant EV71 infectivity titers (P = 0.698 or P = 0.106, respectively). P246A-EV71 and S243A/P243A-EV71 had slightly lower EV71 infectivity titers (P = 0.030 or P = 0.038, respectively) (Fig. 6A). This finding indicated that a proline residue located in the H-I loop acts in the interaction of CypA and EV71, leading to the correct conformation of viral capsid and further virus uncoating.

It is notable that the supernatant infectivity titers of S243P-EV71 were 1.75 log_10_ lower than those of wt-EV71 (P =  0.01, Fig. 6A). The intracellular growth curve showed that S243P-EV71 growth is also slower than that of wt-EV71 (Fig. 6C). However, when we infected RD cells with S243P-EV71 and wt-EV71 viruses under a 5 µM HL051001P2 treatment, we found that the growth of S243P-EV71 was much better than that of wt-EV71 (Fig. 6D). This finding is consistent with the results of the infection by wt-EV71 or S243P-EV71 in RD-sh-CypA cells (Figs. 2C and 2D). Together, these results suggested that replacing S243-VP1 with a proline residue decreased viral fitness but conferred resistance to CypA inhibitors or caused a CypA loss of function.

## Discussion

**Figure 7:**
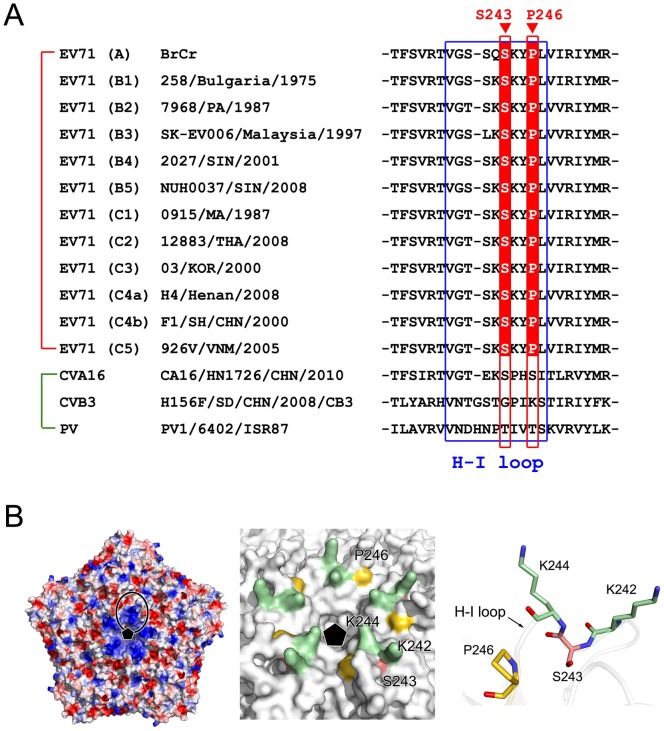
The active site of CypA in EV71 VP1. **(A)** A sequence comparison of the H-I loop in VP1 for the different EV71 strains CVA16, CVB3, and PV. The H-I loop is indicated by a blue frame. The drug-resistant site (VP1-S243) and the potential CypA functional site (VP1-P266) in the H-I loop of EV71 VP1 are also indicated. **(B)** The location of the CypA functional site. Left panel, the electrostatic potential surface of five icosahedral asymmetric units (PDB code: 4AED). Negatively charged surfaces are shown in red, and positively charged areas are shown in blue. The H-I loop region was framed out. The fivefold axis is indicated by a black pentamer. In the middle panel, the molecular surfaces are around the fivefold axis of symmetry. VP1-K242 and -K244, VP1-S243, and VP1-P266 are colored green, red and gold, respectively. Right panel, detailed structure of the H-I loop of EV71 VP1-K242 and -K244, VP1-S243, and VP1-P266. The VP1 molecule is drawn as a transparent cartoon, and the specific residues are shown as colored sticks.

The results we report here demonstrate that the CypA host factor played a crucial role in the uncoating process during the entry step of EV71 infection, and the action site of CypA was mapped to the H-I loop of capsid protein VP1. An analysis of all EV71 sequences in GenBank showed that the action position of CypA in the EV71 VP1 H-I loop was strictly conserved in all EV71 genotypes and stains (Fig. 7A), either in the protein sequence or the gene codon. However, a comparison of several representative strains of Coxsackie virus (CV), poliovirus (PV) and EV suggest that this position is not conserved among EV71, CVA16, CVB3 and PV (Fig. 7A). The dependence of the proliferation of other enteroviruses or picornaviruses on the host factors must be further defined.

The crystal structure of mature EV71 particles [32] revealed that the VP1 H-I loop is a mostly solvent-exposed region at the surface of the virus particle (Fig. 7B) and usually functions in receptor binding or uncoating. Among all reported EV71 functional receptors, SCARB2 is the only one that can mediate both attachment to the host cell and uncoating [21]. PSGL-1 cannot induce the conversion from mature 160S particles to other forms during the viral uncoating process [21]. In a recent result, Nishimur *et al*. reported that the H-I loop of VP1 plays an essential role in EV71 recognition through one of its functional receptors, namely PSGL-1, and they demonstrated that the substitution of VP1-K242 and K244, which are located in the VP1 H-I loop, significantly attenuated virus binding to PSGL-1 [37]. They also indicated that the VP1 E145 residue modulates the orientation of VP1 K244 and thus regulates the exposure of the positively charged lysine side chain, which in turn regulates receptor binding [37]. Moreover, Tan *et al*. showed that EV71 binds to heparan sulfate on the cell surface, and they suggested that heparan sulfate may bind to the positively charged amino acids (including VP1-K242, K244, and R161) that form a cluster around the five-fold symmetry axis [26]. These findings suggested that the lysine residues at the VP1-242 and 244 positions play essential roles in the binding of the EV71 virus to variable receptors. Interestingly, these two lysine residues are surprisingly very close to the CypA action site in the VP1 H-I loop, which is VP1-S243 (Fig. 7B). In a very recent result, Lee *et al*. reported an anti-EV71 neutralizing antibody called MA28-7, which has epitopes at the fivefold vertex that cover the VP1 H-I loop [38]; this study supports the critical role of the correct H-I loop conformation in the EV71 entry step. By contrast, the binding site of the EV71 virion to SCARB2 was mapped at a canyon of VP1 around residue Q172 [21], which is far away from S243-VP1. We observed that EV71 virion binding to SCARB2 was not enhanced, but slightly decreased by CypA treatment, suggesting that the CypA function in SCARB2-mediated EV71 entry could be more complicated. Taken together, we propose that CypA plays a role as an uncoating regulator by altering the conformation of the H-I loop in VP1 during the EV71 entry through the PSGL-1 or HS-mediated pathway, and CypA has different impacts on the entry of EV71 through various functional receptors.

Another interesting observation is that CypA mediated EV71 uncoating most distinctly at pH 6.0, but not at pH 5.5 and 6.0. During virus internalization, endosome acidification increases during maturation, at values ranging from pH 6.8 to 6.1 in early endosomes to pH 6.0 to 4.8 in late endosomes [39], [40]. Because SCARB2 was previously shown to mediate EV71 uncoating most efficiently at pH 5.6 [21], we propose that CypA acts to mediate EV71 uncoating before SCARB2 during the maturation of late endosomes. With the increasing maturation of late endosomes and acidification, the EV71 uncoating regulator transfers from CypA to the next one, namely SCARB2. A similar observation was also found for the CypA study in HPV entry. CypA treatment induced the release of capsid protein L1 from L2 in a pH-dependent manner, in which L1 dissociation from L2 was most efficient at pH 6.0, less efficient at pH 7.4, and undetectable at pH 5.5 and 8.0 [17], suggesting a complicated process during virus uncoating in endosomes.

Furthermore, CypA showed complicated impacts on the HIV-1 life cycle; CypA is necessary for HIV-1 infection [1], [41], [42] but also blocks HIV-1 uncoating [43], as revealed in previous reports. We cannot simply exclude the possibility that CypA may not only be associated with EV71 entry but might also affect other intracellular steps of EV71 protein translation, assembly or secretion in addition to its effects on the entry step. In our results, we actually found that the S243P-EV71 virus proliferated more slowly than the wt-EV71 without CypA inhibitor treatment, although the S243P mutant in VP1 can enhance the interaction between virions and CypA. Moreover, when we infected RD-sh-CypA cells with wt-EV71, although the intracellular genome RNA and VP1 protein level were much lower than that of wt-EV71-infected RD-sh-control cells, the supernatant virus titer exhibited no significant difference. Interestingly, the downregulation of CypB did not attenuate but actually increased EV71 RNA replication and VP1 expression (Figs. 2C and 2D), and the expression of endogenous CypB was also upregulated by EV71 infection (Fig. 2H). Moreover, the S243P mutant recovered both the RNA replication and protein expression of EV71 in RD-sh-CypA cells, but presented discrepancies in RD-sh-CypB cells, i.e., slightly increased RNA replication but decreased protein expression (Figs. 2C and 2E). Interestingly, two recent works revealed that Cyps inhibited the proliferation of HIV-1 virus, which is in opposition to the previously identified positive function of Cyps in the HIV-1 life cycle [9], [44], [45]. All these findings indicated that Cyps may have multiple functions in the EV71 life cycle and may have additional (or opposing) effects on viral assembly and secretion. This finding requires further validation.

The work we describe here highlights the new function of CypA as an uncoating regulator for EV71 proliferation by facilitating the conformational shift of the VP1 H-I loop. Our results significantly increase our understanding of virus-host interactions and provide an additional target of action for CsA-derived antivirals without immunosuppressive activity that are currently in clinical trials for treating EV71-infection.

## Materials and Methods

### Cell lines, viruses and antibodies

RD cells (a human embryonal rhabdomyosarcoma cell line) were purchased from ATCC and the Huh7.5.1 cells were kindly given by Jin Zhong (Institute Pasteur of Shanghai, Chinese Academy of Science). The cells were grown in Dulbecco's modified Eagle's medium (DMEM) (GIBCO) supplemented with 10% fetal bovine serum (FBS) (GIBCO) at 37°C in a humidified incubator with 5% CO_2_. The plasmids containing human EV71 strain AnHui1 (GQ994988.1) and BrCr (U22521) were kindly provided by Prof. Bo Zhang from the Wuhan Institute of Virology. Plasmids containing human EV71 strain SK-EV006 (AB469182.1) and EV71-GFP, which contains a GFP reporter gene that is inserted into the SK-EV006 genome, were donated by Prof. Satoshi Koike (Tokyo Metropolitan Institute of Medical Science). The plasmid with EV71 subgenomic replicon RNA was given by Prof. Wenhui Li (National Institute of Biological Sciences [21]). The pNL4-3 plasmid was given by Prof. Linqi Zhang (School of Medicine, Tsinghua University). The EV71 viruses were amplified in RD cells, quantified by making a determination of the 50% tissue culture infective dose (TCID_50_) per 1 ml in RD cells as previously described [46], and used for all experiments.

A mouse anti-EV71 monoclonal antibody against VP1 (Abcam, 10F0, cat #ab36367) was used to detect the virus in all experiments. Rabbit anti-CypA monoclonal antibody (cat #ab41684-100) and rabbit anti-CypB monoclonal antibody (cat #ab16045) were purchased from Abcam. The anti-GAPDH monoclonal antibody and anti-GST monoclonal antibody were purchased from JiaMei, China. The secondary antibodies used for western blot analysis and immunofluorescence were purchased from Southern Biotech (HRP-conjugated goat anti-mouse IgG(H+L)), CoWin Bioscience (HRP-conjugated goat anti-rabbit IgG), Santa Cruz (PE-conjugated goat anti-mouse IgG), and Life Technologies (donkey anti-rat IgG (H+L)).

### Inhibitors and reagents

The cyclophilin A inhibitor known as CsA was purchased from Sigma, and HL051001P2 compound was generously provided by Prof. Jian Li [28]. The inhibitors were initially dissolved in DMSO, and stock solutions were stored at −20°C. Immediately before addition, these compounds were diluted to the desired concentrations in DMEM with 10% FBS.

TRIzol reagent and a Super Script III First-strand Synthesis System for RT-PCR kit were purchased from Invitrogen. A MEGA script T7 High Yield Transcription kit was purchased from Ambion. A QuantiTect SYBR Green RT-PCR kit was purchased from Qiagen. A cell viability and proliferation assay (WST-1) was purchased from Roche.

### Generating the EV71 infectious virus and EV71 subgenomic replicon

RNA transcripts and the EV71 subgenomic replicon were obtained by using the MEGA script T7 High Yield Transcription kit (Ambion), and the DNA that was linearized by *Sal*I or *Xbal*I (NEB) digestion was used as a template according to the manufacturer's protocol. *In vitro* transcribed RNA was transfected into RD cell monolayers in 100 mm × 20 mm dishes with Lipofectamine 2000 (Invitrogen), and the cells were then incubated at 37°C in 10 ml DMEM containing 10% FBS per dish. The cytopathic effects (CPE) of RD cells were observed at 24 h post transfection. When 90% of the cells exhibited CPE, the cell supernatants were then collected by centrifugation at 4,000 rpm for 5 min, and the target viruses were stored at -80°C.

### Virus titration

The virus titers were determined by using endpoint dilution assays (EPDA), with focus-forming units (ffu) as the read-out [47]. In brief, the measurement was performed by seeding 1×10^4^ RD cells per well in 96-well microtiter plates. After overnight culture, the EV71 viruses were serially diluted 10-fold with DMEM containing 10% FBS (10^−1^- to 10^−8^-fold dilutions) and added to RD cell. The plates were then incubated at 37°C in 5% CO_2_. CPE was observed under the microscope after 3 to 4 days post infection or the GFP expression level was monitored under a fluorescence microscope after 3 days post infection. The virus titer, which was expressed as the TCID_50_, was determined by EPDA.

### RNA analysis

Total cellular RNA was isolated with TRIzol reagent according to standard protocols. The following primer sequences were used for qRT-PCR: GAPDH, forward primer 5′-CCCACTCCTCCACCTTTGACG-3′, reverse primer 5′-CACCACCCTGTTGCTGTAGCCA-3′, EV71 5′-UTR forward primer 5′-TGAATGCGGCTAATCCCAACT-3′, and reverse primer 5′-AAGAAACACGGACACCCAAA G- 3′. qRT-PCR was performed with a QuantiTect SYBR Green RT-PCR kit (Qiagen), and the EV71 and GAPDH transcript levels were determined by ΔΔCT methods.

To determine the amount of purified EV71 virions, viral RNA was extracted from 50 µl of PBS buffer containing EV71 virions by using TRIzol LS reagent (Invitrogen). To determine the amount of EV71 virions in the CsCl fractions, viral RNA was extracted from 125 µl of the CsCl fraction containing EV71 virions with an additional 125 µl of nuclease-free water by TRIzol LS reagent (Invitrogen). A pUC18-EV71AH1 plasmid was used as a standard sample to generate a standard curve ranging from 10^11^–10^3^ copies/ml. EV71 RNA copies were quantified by using the QuantiTect SYBR Green RT-PCR kit (Qiagen).

### A quantitative RT-PCR (qRT-PCR)-based infection assay

The antiviral activities of the compounds were determined by using a qRT-PCR-based assay with the EV71 virus and RD cells. In brief, 100,000 RD cells were seeded in each well of the 24-well tissue culture plates and allowed to attach in complete culture medium overnight. The culture medium was replaced with medium containing serially diluted compounds in the presence of 10% FBS and 0.5% DMSO. After 6 h, the RD cells were infected with EV71 at the multiplicities of infection (MOIs) indicated in the figure legends, and the compounds were added at the indicated concentrations. Total cellular RNA was isolated by using TRIZOL reagent according to standard protocols at 24 hpi. The qRT-PCR assay was performed as described above. The EV71 and GAPDH transcript levels were determined by ΔΔCT method. The IC_50_ value represents the concentration of the compound at which the EV71 RNA level in the RD cells was reduced by 50%.

To monitor the cytotoxic effects of the compounds, the viability of the RD cells was determined after 24 h of compound treatment; the viability was determined in 96-well tissue culture plates by using cell proliferation reagent WST-1 (Roche). Each data point represents the average of three replicates. The EC_50_ and cytotoxicity values were plotted by using GraphPad Prism software.

### Selecting CypA inhibitor-resistant viruses

RD cells were seeded at 5×10^5^ cells/well in 6-well plates. On the following day, the medium was removed and replaced with DMEM containing 10% FBS and 11.4 µM HL051001P2; 0.5% DMSO was used as a control. After 6 h, EV71 strain AnHui1 was used to infect RD cells at an MOI of 0.1 in complete medium containing the inhibitors. Over the course of selection, the RD cells were split when they reached 70–90% confluence. Fresh complete medium containing inhibitors was added when the cell cultures were split. Viral replication in the presence of compound HL051001P2 was monitored by determining the cytopathic effects (CPE) at each passage. The viruses demonstrated apparent CPEs after approximately 5 to 7 days after EV71 infection in the medium containing inhibitors or after 2 days in the medium containing 0.5% DMSO. The cell supernatants were then collected following centrifugation at 4,000 g for 5 min and were stored at −80°C as EV71-P2 (passage 2) virus. The RD cells were then treated with cyclophilin A inhibitors for 6 h and were infected with the EV71-P2 virus under the same conditions described above. The experiment was repeated for 6 cycles, and the cell supernatants were collected as EV71-P6 (passage 6) virus. The RD cells were lysed with TRIzol reagent.

### Identifying CypA inhibitor-resistant mutations

For the EV71 RNA resistance mutation analysis, cellular RNA extraction was performed by using TRIzol reagent (Invitrogen) according to the manufacturer's instructions. For reverse transcription PCR, first strand cDNA was synthesized with a gene-specific primer (5′- ACCCCCACCAGTCACATTCACG- 3′), and the Super Script III First-strand Synthesis System for RT-PCR kit (Invitrogen) was used according to the manufacturer's instructions. The EV71 protein coding region of the genome was amplified by PCR in 5 short fragments as follows: fragment 1 (EV71-718-sense 5′-ATCTTGACCCTTAACACAGC-3′, EV71-2046-anti 5′-GACCATTGGGTGTAGTACCC-3′), fragment 2 (EV71-1975-sense 5′-CGATCCTGGGCGAAGTGGAC-3′, EV71-3345-anti 5′-TGTTGTCCAAATTTCCCAAG-3′), fragment 3 (EV71-3248-sense 5′-TACCTATTCAAAGCCAACCC-3′, EV71-4643-anti 5′-ATAAAGACATATCCTTGCCG-3′), fragment 4 (EV71-4569-sense 5′-ACGGCTACAAGCAACAGGTG-3′, EV71-6044-anti 5′-TTCCCTCGAAGATATCATGG-3′), and fragment 5 (EV71-5972-sense 5′-GGAAGGCTCAACATCAATGG-3′, EV71-7345-anti 5′-GGGTTGAGGTGTGTATAGCC-3′).

The short RT-PCR products of the resistant EV71 virus or the control EV71 virus were ligated into the TA cloning vector PMD18-T (Takara). Multiple individual bacterial colonies were isolated for each time point, and the purified plasmid DNA was sequenced. The sequences were aligned with Sequencher 5.0 and BioEdit software.

### Generating an EV71 mutant recombinant virus

The mutant EV71 recombinant virus clone was constructed on the basis of the pUC18-EV71AH1 plasmid, which contained the original full-length EV71 AnHui1 strain. Site-directed mutagenesis was performed with a QuikChange Lighting Site-Directed Mutagenesis kit (Stratagene). The mutagenic primers were designed as follows: S243P-EV71(5′-GTGGGGACCTCCAAGCCCAAGTACCCTTTAG- 3′), S243A-EV71(5′-GTGGGGACCTCCAAGGCCAAGTACCCTTTAG-3′), P246A-EV71(5′-GGACCTCCAAGTCCAAGTACGCTTTAGTGGTTAGAATTTACATG-3′), S243P/P246A-EV71(5′-GTGGGGACCTCCAAGCCCAAGTACGCTTTAGTGGTTAGA ATTTAC-3′), and S243A/P246A-EV71(5′-GTGGGGACCTCCAAGGCCAAGTACGCTTTA GTGGTTAGAATTTAC-3′). The constructs were confirmed by sequencing.

### Generating stable knockdown cell lines

The CypA and CypB stable knockdown RD or Huh7.5.1 cell lines were produced as previously described, with some modifications [12]. The following shRNA sequences were used in this study: NC, 5′-TTCTCCGAACGTGTGTCACGTTTC-3′; CypA, 5′-CTGGATTGCAGAGTTAAGTTTA-3′; and CypB, 5′-GCCGGGTGATCTTTGGTCTCTT -3′. shRNA recombinant lentiviruses (LV2-NC, LV-CypA, LV-CypB) were produced by Shanghai GenePharma, and the virus titers were determined to be 1×10^8^ TU/ml. RD or Huh7.5.1 cells were infected at an MOI of 10. The shRNA recombinant lentivirus was incubated with 5 µg/ml polybrene to enhance the lentivirus infection. All knockdown cell lines were confirmed at 72 h post infection by western blot analysis. For the stable knockdown cell lines, the RD or Huh7.5.1 cells were incubated in selection medium containing 5 µg/ml puromycin (Invitrogen) beginning 48 h after transduction, and the CypA and CypB knockdowns were stable after approximately two weeks of cell culture.

### Infection and immunofluorescence assays after an siRNA knockdown of Cyps

RD cells or CypA knockdown cells were grown on cover slips until the cells reached 50% confluence; the cells were then infected with the EV71 virus. The cells were washed with PBS at the indicated times post infection and fixed with 4% paraformaldehyde for 15 min at room temperature, washed, and permeabilized with 0.5% Triton X-100 in PBS for 10 min. The cells were then washed and blocked with 1% normal goat serum in PBS for 30 min, followed by a 1 h incubation with primary antibodies (1∶400 dilution) at room temperature. After three washes with PBS, the cells were incubated with FITC- or PE-conjugated secondary antibodies (a 1∶200 dilution) for 1 h. After extensive washing with PBS, the cell nuclei were stained with DAPI. Images were captured by using a confocal microscope (Olympus FluoView FV1000 Confocal Microscope operated by FluoView software). The same microscope settings and exposure times were used within the individual experiments.

### Recombinant protein production

The genes encoding the H-I loop of EV71 VP1 (residues 239-GSSKSKYPL-247), the H-I loop with S243P substitution (residues 239-GSSKPKYPL-247), human CypA and the catalytic-defective mutant CypA H126Q were cloned into the pGEX-6p-1 expression vector with a GST tag fused at the N-terminus according to a general protocol. The accuracy of the insert was verified by sequencing. The plasmids were transformed into *E. coli* BL21 (DE3) cells, and the transformed cells were cultured at 37°C in LB media containing 100 mg/L ampicillin. After the OD_600_ reached 0.5, the culture was cooled to 16°C, and recombinant protein expression was induced. After overnight induction, the cells were harvested by centrifugation. The pellets were then resuspended in lysis buffer containing 20 mM Tris-HCl (pH 7.5) and 150 mM NaCl, followed by homogenization using an ultra-high-pressure cell disrupter (JNBIO, Guangzhou, China) at 4°C. The insoluble material was removed by centrifugation at 20,000 *g*. The supernatant was then loaded twice onto a GST column pre-equilibrated with lysis buffer. After loading, the GST column was washed with at least 5 column volumes of lysis buffer to remove the unbound protein. The beads containing recombinant GST-1S, GST-1P, GST-CypA or GST protein were added to Eppendorf tubes and stored at −80°C until use. To obtain recombinant human CypA without a GST tag, the GST tag on CypA was removed by overnight incubation with PreScission Protease, and the target proteins were eluted with lysis buffer. The eluted target proteins were further purified by Superdex-75 gel filtration chromatography (GE Healthcare) to remove any contamination. The fractions were analyzed with SDS-PAGE, and the final purity was over 95%.

### Immunoblotting and pull-down assays

For the immunoblot analysis, the cells were lysed in a lysis buffer containing 50 mM Tris-HCl (pH 8.0), 150 mM NaCl, 1% Nonidet P-40, 0.1% SDS, 2 mM EDTA, and protease inhibitors; the protein concentrations of the lysates were determined with a spectrophotometer. The proteins were resolved by sodium dodecyl sulfate polyacrylamide gel electrophoresis (SDS-PAGE) and transferred to nitrocellulose membranes (Millipore). The membranes were blocked for 4 h with 5% nonfat dry milk solution in Tris-buffered saline. The membranes were then blotted with specific primary antibodies, followed by incubation with secondary antibodies conjugated to horseradish peroxidase. The proteins were visualized by chemiluminescence by using a Clarity Western ECL Substrate (BIO-RAD). To allow the pull-down assays to detect the interaction between CypA and the EV71 virion, we incubated 200 µl of the EV71 strain (AnHui1, SK-EV006, S243A/P246A-EV71 and BrCr) (2×107 TICD50) with 50 µl of glutathione-sepharose beads containing GST (200 µg) or GST-CypA (200 µg) in 300 µl of immunoprecipitation buffer (50 mM Tris-HCl, pH 8.0, 150 mM NaCl, 1% Nonidet P-40, 2 mM EDTA, and protease inhibitors) overnight at 4°C. The beads were then washed three times with PBS, and the complexes were eluted from the glutathione-sepharose beads with reduced glutathione (GSH) solution. We then moved the supernatant to new Eppendorf tubes and added SDS loading buffer. We then incubated the supernatants in boiling water for 5 min and subjected the samples to 12% SDS-PAGE followed by western blot analysis with mouse antibodies against EV71-VP1 or GST. To detect the interaction between CypA and GST-1S or GST-1P, recombinant human CypA was expressed, purified, and concentrated to 20 mg/ml. Recombinant human CypA (200 µg) was incubated with 50 µl of glutathione-sepharose beads containing GST (200 µg), GST-1S (200 µg), or GST-1P (200 µg) in 300 µl of immunoprecipitation buffer as described above, and the reactions were incubated overnight at 4°C. We then washed the beads three times with PBS and eluted the complexes from the glutathione-sepharose beads with reduced GSH solution. The samples were then subjected to western blot analysis, as described above, by using mouse antibodies against GST or CypA. The bands were quantified by ImageJ software.

### Time-of-addition assay

The time of addition effect was examined for HL051001P2. RD cells (1.5×10^5^ per well in 500 µl of 10% FBS-DMEM medium) were cultured at 37°C under 5% CO_2_ in 24-well plates overnight. The cells were subsequently treated with 5 µM HL051001P2 either concurrent with the wt-EV71 (0 h) at an MOI of 50 or at intervals of −6, −4, −2, 0, 4, 6, and 8 hpi. After incubating at 37°C for 12 h, the antiviral activity was determined by measuring the percentage of EV71 RNA from the untreated control cells, and the mRNA level of GAPDH was used as an internal control. The supernatant virus titer, which was expressed as the TCID_50_, was determined by EPDA.

### A binding assay of the virus to the host cells

The virus binding assay was performed by using a previously reported protocol with some modifications [48]. In brief, RD cells were seeded at 1×10^5^ RD cells/well in 24-well plates. The following day, the culture medium was removed and the cells were washed once with cold phosphate-buffered saline (PBS). After that, 500 µl of binding buffer (PBS containing 1% BSA and 0.1% sodium azide) was added to the cells on ice and incubated for 10 min; the supernatant was subsequently removed from the cells. The EV71 stocks (Strain AnHui1) (10^8^ TCID_50_/ml) were prepared as previously mentioned. The EV71 virus was diluted in 500 µl of DMEM complete medium (dilution fold = 1:10 [5×10^6^ TCID_50_], or 1:20 [2.5×10^6^ TCID_50_]) per well and added to the cells. After 1 h of incubation on ice, the unbound virus was removed by three wash steps with 500 µl of PBS, and the cells were lysed in the wells with 500 µl of TRIzol. Viral RNA was extracted and detected by qRT-PCR. The virus binding assays were systematically performed in duplicate, and two individual experiments were performed for each condition.

### Binding assays of viruses with HS, PSGL-1 or SCARB2

The virus binding assay was performed according to a previously reported protocol [23], [26]. To detect the influence of CypA on the HS and EV71 virus interaction, 4 ml of EV71 strain AnHui1 (4 ×10^8^ TICD_50_) was incubated with or without CypA at 4°C for 2 h. The supernatant was added to a 1 ml HiTrap Heparin HP column (GE Healthcare, Sweden) that was previously equilibrated with binding buffer (0.02 M Tris-HCl and 0.14 M NaCl [pH 7.4]) at a flow rate of approximately 0.5 ml/min. After loading, the two columns were washed with at least 5 column volumes of binding buffer to remove the unbound virus. The bound viral particles were eluted by using elution buffer (0.02 M Tris-HCl and 2 M NaCl [pH 7.4]). Fractions of 1 ml were collected, and the EV71 RNA was isolated and quantified by using the qRT-PCR method described above. Purified CypA was also uploaded to the same HiTrap Heparin HP and did not show any detectable interaction between CypA and the column (data not shown).

To detect the CypA influence on the PSGL-1 and EV71 virus interaction, EV71 strain AnHui1 (2 ×10^7^ TICD_50_) was incubated with or without recombinant human CypA (4 µg) in 200 µl of DMEM for 2 h at 4°C. Human PSGL-1-Fc (3 µg, R&D Systems) or human IgG VRC01 Fc (3 µg) (a control), were then added to the assays, and the reactions were incubated for 1 h at 4°C. Protein G-agarose (50 µl, Roche) was then added to the mixture, and the tubes were shaken overnight at 4°C.

To detect the CypA treatment effect of the SCARB2 and EV71 virus interaction, EV71 strain AnHui1 (2×10^7^ TICD_50_) was incubated with or without recombinant human CypA (6 µg) in 200 µl of DMEM for 2 h at 4°C. SCARB2 at a 5 µg quantity was added to the assays, and the reactions were incubated for 1 h at 4°C. Ni-NTA beads (50 µl) were then added to the mixture, and the tubes were shaken overnight at 4°C. All the beads were washed three times with PBS and then eluted with 100 µl of elution buffer containing 20 mM Tris-HCl, pH 8.0, 500 mM NaCl and 1 M imidazole. The EV71 RNA in the supernatant was isolated by TRIzol LS reagent and quantified by using the qRT-PCR method described above.

### Purifying wt-EV71 by ultracentrifugation

The purification of wt-EV71 virions was performed by using a previously reported protocol with modifications [21]. In brief, RD cells in five T175 cell culture flasks were infected with EV71 (Anhui1 strain) at an MOI of 0.1 and cultured in 10% FBS. When 90% of the cells exhibited CPE, the supernatant was collected and concentrated by filtration through a 100 kDa-cutoff centrifugal filter (Millipore). The concentrated virus was mixed with 1.4 g/ml CsCl at a volume ration of 1∶4 and loaded on the middle of a CsCl gradient (1.1 g/ml, 1.2 g/ml, 1.3 g/ml, 1.4 g/ml, and 1.5 g/ml, discontinuously) followed by ultracentrifugation at 41,000 rpm for 10 h at 4°C in a Beckman SW41Ti rotor. After being dialyzed with PBS, the purified EV71 virus was quantified and stored at -80°C. The RNA copies of purified EV71 virus was quantified as previously described.

### A virion flotation assay of the CsCl density gradient centrifugation

Fifty µl of purified EV71 virus (1×10^10^ genome copies) was incubated with 20 µg of purified wt CypA or catalytic-defective mutant CypA H126Q in PBS containing 0.5% BSA in a total volume of 200 µl. For the low pH treatments, HCl was added to the mixture to bring the pH values to 5.5, 6.0 and 6.5. The mixture was then incubated at 37°C for 4 h and subsequently applied to a 1.1-1.5 g/ml discontinuous CsCl gradient, which was then ultracentrifuged at 41,000 rpm for 10 h at 4°C in a Beckman SW41Ti rotor. The samples were then analyzed by qRT-PCR. The transition states of viral particles during uncoating was performed *in vitro* with native 160S virions by heating for 10 min in a low salt buffer containing 4 mM CaCl_2_, 20 mM HEPES, pH 7.4 at 61°C and 68°C.

## Supporting Information

Figure S1 **Recombinant CypA catalyzed the** ***cis*** **-** ***trans*** **reaction of chemically synthesized peptides.** The shifts in NMR spectra indicated the conformational peptidyl-proline change [49]–[51]. At 500 MHz ^1^H NMR spectra of peptides at 10°C and pH 6.0, the concentration of each peptide is 2 mM. 1S without CypA treatment **(A)**, 1P without CypA treatment **(C)**, or 1S **(B)** and 1P **(D)** with CypA treatment (the concentration was 20 µM) for 1 h at 4°C. The signal marked here is from the ortho-proton signals from the *p*-nitroanilide of peptides, which represents the signals from the *cis* isomer (the chemical shift is 7.32 ppm) or the *trans* isomer (the chemical shift is approximately 7.42 ppm). The integral area of the respective peaks is used as the upper numerical value. The 1S peptide sequence is GSSKSKYPL, and 1P peptide sequence is GSSKPKYPL.
(TIF)

Table S1 **VP1 amino acid changes that emerged during selection with compound HL051001P2.** R-1, R-2, and R-3 represent three individual selection experiments for CypA inhibitor-resistant virus.
(DOC)

Table S2 **HL051001P2 or CsA resistance levels for site-directed changes that were engineered into the EV71 virus.** The S243P substitution in EV71 VP1 confers resistance to HL051001P2. The RD cells were incubated for 6 h with various concentrations of compound HL051001P2 (0.06 to 20 µM) and CsA (0.06 to 20 µM) and then infected with wt-EV71 virus Anhui1 or S243P EV71 at an MOI of 1. The EV71 RNA levels were quantified by RT-qPCR. Each data point represents the average for three replicates.
(DOC)

Text S1 **Supporting information for the NMR measurement of CypA activity.** The PPlase activity of recombinant activity was measured as in a previously described NMR method [49]–[51]. In brief, the recombinant CypA protein used in the NMR experiments was diafiltered into 50 mM phosphate buffer, pH 6.0, and concentrated at a stock concentration of 1 mM. The substrate peptides were chemically synthesized and dissolved to a concentration of 2 mM in buffer containing 50 mM sodium phosphate, pH 6.0. The recombinant CypA protein (at a final concentration of 20 µM) was added to the substrate peptides and the mixture was incubated for 1 h at 4°C. A 10% D_2_O solution was used as a lock sample in the NMR spectrometer. Aliquots were added to the 5 mm NMR tube. ^1^H NMR measurements were performed at 500.13 MHz, 10°C, on a Bruker-600 MHz NMR spectrometer, and the acquired data were processed with MestReNova software.
(DOC)
