# Supplementary figures and images for: Cyclophilin A Associates with Enterovirus-71 Virus Capsid and Plays an Essential Role in Viral Infection as an Uncoating Regulator
Source: PLoS Pathog. 2014 Oct 2;10(10):e1004422. doi: 10.1371/journal.ppat.1004422 (PMC4183573; doi:10.1371/journal.ppat.1004422)

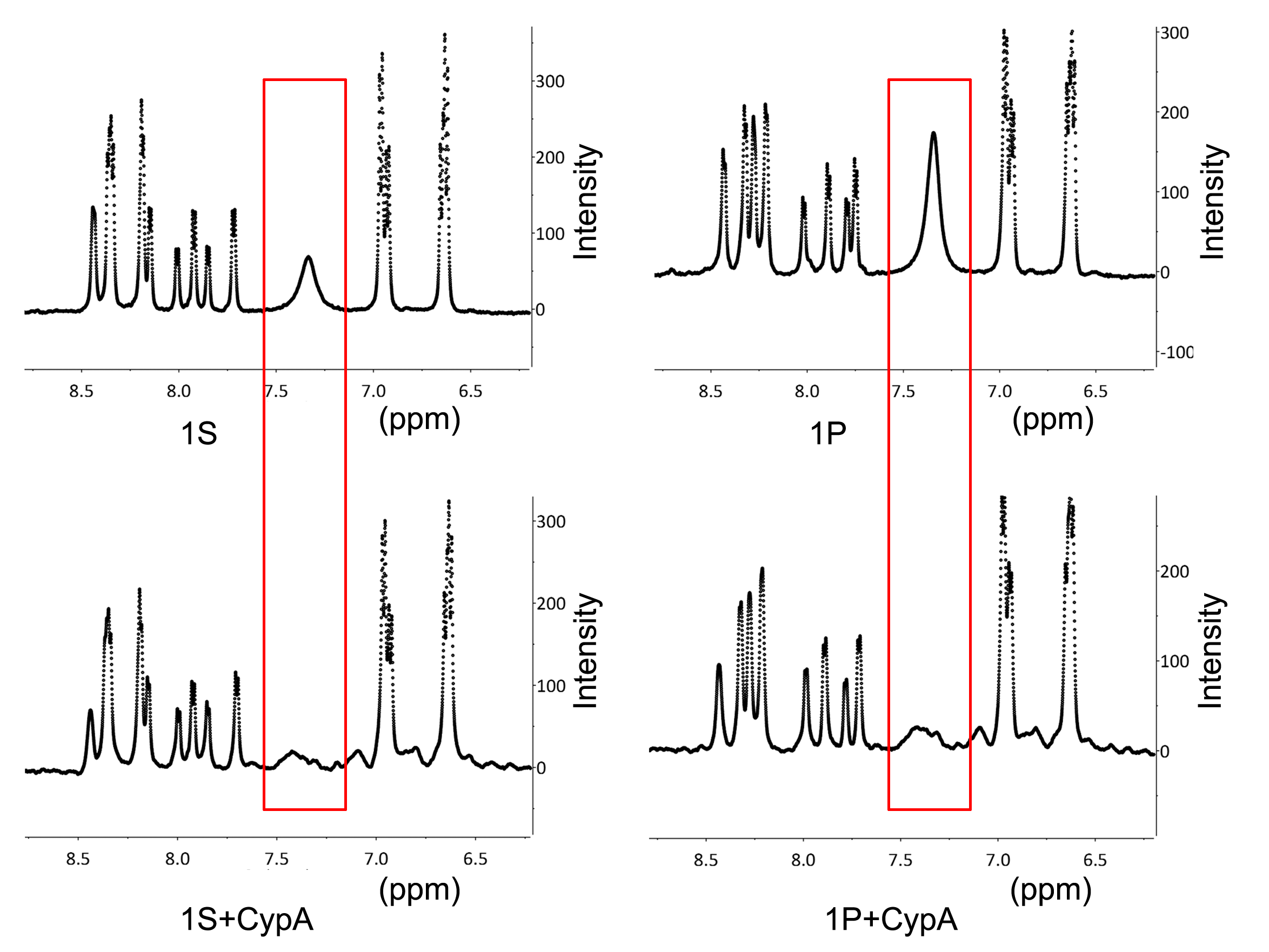

Supplement: Figure S1 — Recombinant CypA catalyzed the cis - trans reaction of chemically synthesized peptides. The shifts in NMR spectra indicated the conformational peptidyl-proline change [49]–[51]. At 500 MHz 1H NMR spectra of peptides at 10°C and pH 6.0, the concentration of each peptide is 2 mM. 1S without CypA treatment (A), 1P without CypA treatment (C), or 1S (B) and 1P (D) with CypA treatment (the concentration was 20 µM) for 1 h at 4°C. The signal marked here is from the ortho-proton signals from the p-nitroanilide of peptides, which represents the signals from the cis isomer (the chemical shift is 7.32 ppm) or the trans isomer (the chemical shift is approximately 7.42 ppm). The integral area of the respective peaks is used as the upper numerical value. The 1S peptide sequence is GSSKSKYPL, and 1P peptide sequence is GSSKPKYPL. (TIF) [file ppat.1004422.s001.tif]
