# Supplementary material for: Cyclophilin A Associates with Enterovirus-71 Virus Capsid and Plays an Essential Role in Viral Infection as an Uncoating Regulator
Source: PLoS Pathog. 2014 Oct 2;10(10):e1004422. doi: 10.1371/journal.ppat.1004422 (PMC4183573; doi:10.1371/journal.ppat.1004422)
Supplement: Table S1 — VP1 amino acid changes that emerged during selection with compound HL051001P2. R-1, R-2, and R-3 represent three individual selection experiments for CypA inhibitor-resistant virus. (DOC) [file ppat.1004422.s002.doc]

# Supplementary Tables

## Table S1. VP1 amino acid changes that emerged during selection with compound HL051001P2.

| **Amino Acid Substitutions** | **emergence chance** | | | |
| --- | --- | --- | --- | --- |
|  | Treated without or with compound HL051001P2 at 11.4 μM | | | |
|  | 0.5% DMSO | R-1 | R-2 | R-3 |
| S243P | 0/4 | 3/3 | 6/6 | 4/4 |

R-1, R-2, and R-3 represent three individual selection experiments for CypA inhibitor-resistant virus.
