# Supplementary material for: Cyclophilin A Associates with Enterovirus-71 Virus Capsid and Plays an Essential Role in Viral Infection as an Uncoating Regulator
Source: PLoS Pathog. 2014 Oct 2;10(10):e1004422. doi: 10.1371/journal.ppat.1004422 (PMC4183573; doi:10.1371/journal.ppat.1004422)
Supplement: Table S2 — HL051001P2 or CsA resistance levels for site-directed changes that were engineered into the EV71 virus. The S243P substitution in EV71 VP1 confers resistance to HL051001P2. The RD cells were incubated for 6 h with various concentrations of compound HL051001P2 (0.06 to 20 µM) and CsA (0.06 to 20 µM) and then infected with wt-EV71 virus Anhui1 or S243P EV71 at an MOI of 1. The EV71 RNA levels were quantified by RT-qPCR. Each data point represents the average for three replicates. (DOC) [file ppat.1004422.s003.doc]

## Table S2. HL051001P2 or CsA resistance levels for site-directed changes that were engineered into the EV71 virus.

| Inhibitors | EC_50_ (µM) | | Resistance (fold/wt) |
| --- | --- | --- | --- |
|  | wt EV71 | S243P EV71 |  |
| HL051001P2 | 0.78 ± 0.64 | 3.56 ± 0.91 | 4.56 |
| CsA | 5.10 ± 1.72 | 9.32 ± 1.06 | 1.83 |

The S243P substitution in EV71 VP1 confers resistance to HL051001P2. The RD cells were incubated for 6 h with various concentrations of compound HL051001P2 (0.06 to 20 µM) and CsA (0.06 to 20 µM) and then infected with wt-EV71 virus Anhui1 or S243P EV71 at an MOI of 1. The EV71 RNA levels were quantified by RT-qPCR. Each data point represents the average for three replicates.
