# Supplementary material for: Cyclophilin A Associates with Enterovirus-71 Virus Capsid and Plays an Essential Role in Viral Infection as an Uncoating Regulator
Source: PLoS Pathog. 2014 Oct 2;10(10):e1004422. doi: 10.1371/journal.ppat.1004422 (PMC4183573; doi:10.1371/journal.ppat.1004422)
Supplement: Text S1 — Supporting information for the NMR measurement of CypA activity. The PPlase activity of recombinant activity was measured as in a previously described NMR method [49]–[51]. In brief, the recombinant CypA protein used in the NMR experiments was diafiltered into 50 mM phosphate buffer, pH 6.0, and concentrated at a stock concentration of 1 mM. The substrate peptides were chemically synthesized and dissolved to a concentration of 2 mM in buffer containing 50 mM sodium phosphate, pH 6.0. The recombinant CypA protein (at a final concentration of 20 µM) was added to the substrate peptides and the mixture was incubated for 1 h at 4°C. A 10% D2O solution was used as a lock sample in the NMR spectrometer. Aliquots were added to the 5 mm NMR tube. 1H NMR measurements were performed at 500.13 MHz, 10°C, on a Bruker-600 MHz NMR spectrometer, and the acquired data were processed with MestReNova software. (DOC) [file ppat.1004422.s004.doc]

# Supplementary Text S1

## Text S1. NMR measurement of the activity of CypA

The PPlase activity of recombinant activity was measured as in a previously described NMR method [1-3]. In brief, the recombinant CypA protein used in the NMR experiments was diafiltered into 50 mM phosphate buffer, pH 6.0, and concentrated at a stock concentration of 1 mM. The substrate peptides were chemically synthesized and dissolved to a concentration of 2 mM in buffer containing 50 mM sodium phosphate, pH 6.0. The recombinant CypA protein (at a final concentration of 20 μM) was added to the substrate peptides and the mixture was incubated for 1 h at 4°C. A 10% D_2_O solution was used as a lock sample in the NMR spectrometer. Aliquots were added to the 5 mm NMR tube. ^1^H NMR measurements were performed at 500.13 MHz, 10 °C, on a Bruker-600 MHz NMR spectrometer, and the acquired data were processed with MestReNova software.

# Supplementary References for Text S1

1. Fischer G, Wittmann-Liebold B, Lang K, Kiefhaber T, Schmid FX (1989) Cyclophilin and peptidyl-prolyl cis-trans isomerase are probably identical proteins. Nature 337: 476-478.

2. Galat A, Metcalfe SM (1995) Peptidylproline cis/trans isomerases. Progress in biophysics and molecular biology 63: 67-118.

3. Hubner D, Drakenberg T, Forsen S, Fischer G (1991) Peptidyl-prolyl cis-trans isomerase activity as studied by dynamic proton NMR spectroscopy. FEBS Lett 284: 79-81.
